# Dosimetric analysis and comparison of reduced longitudinal cranial margins of VMAT-IMRT of rectal cancer

**DOI:** 10.1186/s13014-018-1120-0

**Published:** 2018-09-06

**Authors:** Hendrik Dapper, Markus Oechsner, Stefan Münch, Kai Borm, Jan Peeken, Michael Mayinger, Stephanie E. Combs, Daniel Habermehl

**Affiliations:** 1 Department of Radiation Oncology, Klinikum rechts der Isar, TU München, Ismaninger Str. 22, 81675 Munich, Germany; 2 Institute for innovative Radiotherapie (iRT), Helmholtz Zentrum München, Ingolstädter Landstr. 1, Neuherberg, Germany; 3 Deutsches Konsortium für Translationale Krebsforschung (DKTK), Partner Site Munich, Munich, Germany

**Keywords:** Rectal cancer, Reduced cranial margins, Dosimetric quantification, IMRT

## Abstract

**Background:**

The cranial border of the target volume (TV) in rectal cancer patients treated with neoadjuvant chemoradiation (nCRT) is mostly defined at the level of L5/S1. However, current studies have shown that relapse cranially of the target volume after neoadjuvant nCRT and surgery is very rare. A reduction of cranial TV margins could be reasonable to reduce toxicity to the organs at risk (OAR). In this study we compared the dose distribution to the OAR for different cranial longitudinal margins using a dose-volume histogram (DVH) analysis.

**Methods:**

Ten patients with loco regional advanced rectal cancer were analysed retrospectively. All patients were planned for Volumetric Arc Therapy Radiation Therapy (VMAT). Next to the original PTV (PTV0), three new planning target volumes (PTV) were defined for each patient: The PTV0 reduced by 1 cm, 2 cm and 3 cm on cranial extension. For each PTV a treatment plan with a total dose of 50.4 Gy with daily doses of 1.8 Gy was calculated. Dose to the OAR were evaluated and compared.

**Results:**

For the bone marrow, the small bowel and the peritoneal space all clinically relevant relative dose parameters (V10-V50) as well as the Dmedian could be significantly reduced with every cranial target volume reduction of 1 cm. For V10 of the peritoneal space the dose could be nearly halved with a 3 cm shortened TV. After TV reduction of 3 cm also for the urinary bladder a significant dose reduction of the Dmedian could be achieved.

**Conclusions:**

Considering the very low recurrence rates in the TME and IMRT era, the distribution patterns of these relapses as well as the relevant side effects of nCRT, we would agree with existing recommendations of reduction of the cranial target volume in nCRT treated rectal cancer patients.

## Background

Neoadjuvant short term radiation therapy (RT, 5 × 5 Gy) or conventional chemoradiation (CRT) are the standard treatment protocols for patients with locally advanced rectal cancer (UICC-Stage II or III) for potential curative disease of the lower and middle third [1]. Several studies have demonstrated that with n(C)RT loco regional relapse rates can be significantly reduced and sphincter preservation rates in lower carcinoma can be improved [2–4].

In the last decade there have been significant changes in both, surgery and radiation techniques in the treatment of rectal cancer patients. The total mesorectal excision (TME) alone can provide very good local control rates. In some trials, long term local control, especially for T3 N0 tumors, could reach more than 90% compared with conventional operation techniques [4, 5]. But even though local control rates improved with TME alone, not all institutes can achieve such good results and even with excellent surgical technique, neoadjuvant radiotherapy can usually halve the number of loco regional relapse [4, 6]. Furthermore, intensity modulated radiation therapy (IMRT) of the pelvic is more conformal than conventional radiation therapy and achieves better dose sparing of OAR [7–9]. But though modern n(C)RT techniques can effectively improve loco regional control, it is not without side effects. Especially the hematologic and acute gastrointestinal side effects are still relevant and can lead to treatment breaks [10–12].

Radiation contouring guidelines mainly based on data before the TME era, although studies dealing with patterns of relapse have shown that loco regional relapse is mainly below the level of S1-S2 [13], without primary nodal involvement and a negative circumferential resection margin even below S2-S3 [14]. The RTOG Consensus Panel for elective Clinical Target Volumes (CTV) in Anorectal Cancer recommends an inclusion of the presacral space where common iliac vessels bifurcate into external/internal iliacs (approximate boney landmark: sacral promontory) [15]. In case of positive lymph nodes, the current International Consensus Guidelines on Clinical Target Volume Delineation in Rectal Cancer recommend an inclusion of the prescaral space at the level of the bifurcation of the aorta in common iliac arteries or 5 mm above the last positive lymph node into the CTV [16].

It was shown that lowering the cranial CTV margins reduces the small bowel exposure with 3D and IMRT radiotherapy for long course neo-adjuvant treatment [17]. With this study we want to verify to what extent a reduction of different cranial CTV margins in nCRT treated rectal cancer patients leads to positive effects on dose-volume distribution.

## Methods

### Patient characteristics

A total of 10 rectal cancer patients with UICC stadium III (T3N+) treated with a conventional nCRT concept (total dose 50.4 Gy, single dose 1.8 Gy) from 2014 to 2017 in our institution were included in this study. We selected patients with tumors 5–12 cm from the anal verge. All patients received at least one pre-therapeutic MRI, a proctorectoscopy and a planning CT scan. Only those patients were included, where the last macroscopic suspected visible tumor (on primary tumour site or suspected lymph nodes) in pre-treatment MRI could be identified 4 cm below the cranial boarder of the pelvic pre-sacral space (bifurcation of the common iliac arteries). All patients received concomitant chemotherapy. Either 5-Fluoruracil (5FU) on day 1–4 and 29–32 with 1000 mg/qm body surface area (BSA) or Capecitabine 825 mg/qm BSA two times a day, 5 days per week. Patients´ characteristics are shown in Table 1.

**Table 1:** Patient characteristics

Patient	Gender	TNM	Primarius: Localisation from anal verge	Lymph node localisation	Cranial PTV margin
1	f	uT3 cN1b	4–9 cm	fossa obturatoria r.	L5/S1
2	m	uT3 uN1a	5–10 cm	perirectal	L5 sup./post.
3	f	uT3 cN1b	5–10 cm	fasica	L5 sup./post.
4	m	uT3 uN1a	4–7 cm	perirectal	L5 sup./ventral
5	m	uT3 uN2a	5–11 cm	fasica	L5/S1
6	m	uT3 uN1b	6–10 cm	perirectal	L5/S1
7	m	uT2 uN2b	9–12 cm	presacral, perirectal	L4
8	m	cT3 cN2a	6–10 cm	fasica	L4 sup./ventral
9	m	uT3 uN2a	4–9 cm	perirectal	L5 sup./post.
10	f	uT3 cN1a	5–9 cm	perirectal	L5 sup./ventral

*r* right, *sup* superior, *post* posterior, *m* male, *f* female

### Contouring

Contouring and treatment planning was performed using Eclipse 13.0 planning system (Varian Medical Systems, Palo Alto, CA, USA). Contouring was performed on planning CTs with 3 mm slice thickness. All patients were immobilized in prone position. For the definition of the target volumes, we also used MRI-scans and the information of the proctorectoscopy as well as PET-CT/MRI information, if available. The clinical target volume (CTV) definition for standard PTV (PTV0) were based on the recommendations of the RTOG Consensus Panel Contouring Atlas [15].

**Fig. 1:**
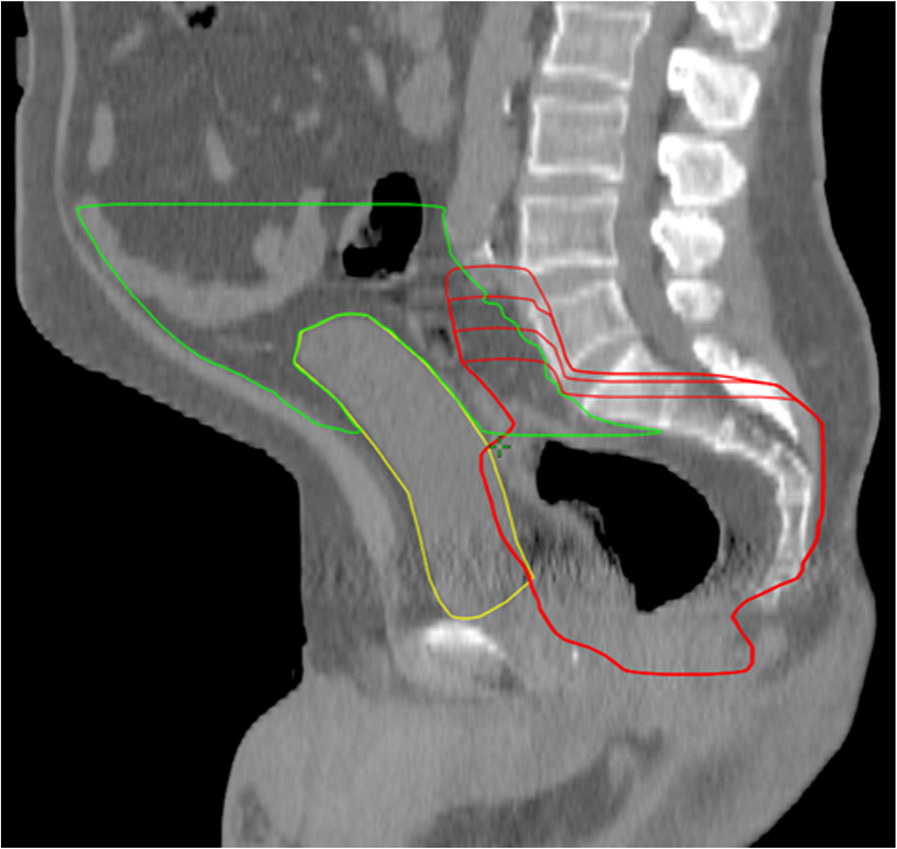
Rectal cancer - cranial radiation target volume reduced by 1, 2 and 3 cm. Red: Original PTV, PTV -1 cm, PTV-2 cm and PTV-3 cm. Original PTV cranial on level of L4/L5 interspace (posterior), PTV - 3 cm on the level of L5/S1. Green: Peritoneal space, yellow: Urinary bladder

Three new clinical target volumes were defined and extended by 1 cm to create the PTVs. First, the original CTV was reduced by 1, 2 and 3 cm (longitudinal) and if necessary, adjusted to bones and vessels. By using 1 cm safety margin of these CTVs, PTVs were defined for each patient: The standard PTV minus 1 cm cranially (PTV-1), the standard PTV minus 2 cm cranially (PTV-2) and the standard PTV minus 3 cm cranially (PTV-3) (Fig. 1). The goal was to keep the shortest PTV (PTV-3) at least 2 cm above the last macroscopically visible primary tumor or lymph node metastases on MRI scan.

The small bowel, as well as the peritoneal space as a surrogate, the urinary bladder, the bone marrow, the femoral heads and the genitals were contoured as OARs on the planning CT-scans. We defined the pelvic bones as they could be identified on planning CT-scan. Cranial lineation was 2.1 cm above PTV0 and caudal lineation was 2.1 cm below the PTV0. The whole of sacral, iliac, ischial and pubic bone as well as the acetabulum and L5 and L4 were included. The small bowel loops were contoured as they could be identified on planning CT scan. The peritoneal space includes the small bowel, the large bowel, peritonealised large bowel and intraperitoneal vessels. The sexual organs as well as the urinary bladder were excluded. Contouring was done almost analogous to Robyn Banerjee et al. 2012 [18]: Superiorly we used 7 slices above the cranial end of the original PTV. Inferiorly it was 3 mm below the lowest identified small bowel loop or on the level of the peritoneal sigmoid colon. The anterior boarder was the abdominal muscles and posterior the vertebral bodies, vena cava and aorta as well as the psoas muscles. Laterally, the boundary was the pelvic side wall and the muscles.

### Treatment planning and evaluation

For all ten patients we optimized four treatment plans for PTV0–3 using Volumetric-modulated arc therapy (VMAT) with two full arcs (358° rotation) and 15 MeV photons. Plans were optimized for treatment with a Varian Clinac DHX linear accelerator (Varian Medical Systems, Palo Alto, CA, USA).

The planning goal was to achieve a homogeneous dose distribution within the PTV and to reduce the dose to OARs, in particular the small bowel, bladder, bone marrow, femoral heads and the genitals.

Dose calculations were performed using the Anisotropic Analytical Algorithm (AAA) and heterogeneity correction. The prescribed dose for the neoadjuvant treatment plans was 50.4 Gy with single doses of 1.8 Gy. All plans were normalized to a median dose of the PTV corresponding to the prescription dose.

To compare dose distribution to the OAR we analysed absolute median dose (Dmedian) and maximum dose (Dmax) on all treatment plans (PTV, PTV-1, PTV-2, PTV-3). For the small bowel loops and the peritoneal space, as a surrogate for the small bowel, we also analysed relative dose parameters (volume receiving 10 Gy, 15 Gy, 20 Gy, 25 Gy, 30 Gy, 35 Gy, 40 Gy, 45 Gy, 50 Gy) for all treatment plans. The volumes receiving 10 Gy, 20 Gy, 30 Gy and 40 Gy (V10 - V40) of the bone marrow and the absolute dose of 65 cc, 100 cc, 180 cc and 830 cc to the peritoneal space were additionally evaluated for all treatment plans.

For all dose parameters of the OAR, a two-sided Wilcoxon test was performed with SPSS 25.0 (SPSS Inc., Chicago, IL, USA) to identify significant differences between the plans for PTV0 and PTV1–3. A *p*-value < 0.05 was considered to indicate statistical significance.

## Results

### Dosimetric analysis

The standard PTV (PTV0) (the original planned target volume) differed in the cranial boarder in the 10 patients. The highest extension was at the level of L4 (vertebral middle), the lowest was at the level of L5/S1 (superior/anterior). In mean, it was in the longitudinal middle of L5. The cranial margin of PTV minus 3 cm (PTV-3) varies between the level of L5/S1 (posterior) and the level of S2 (inferior/anterior). Patient characteristics are found in Table 1.

**Fig. 2:**
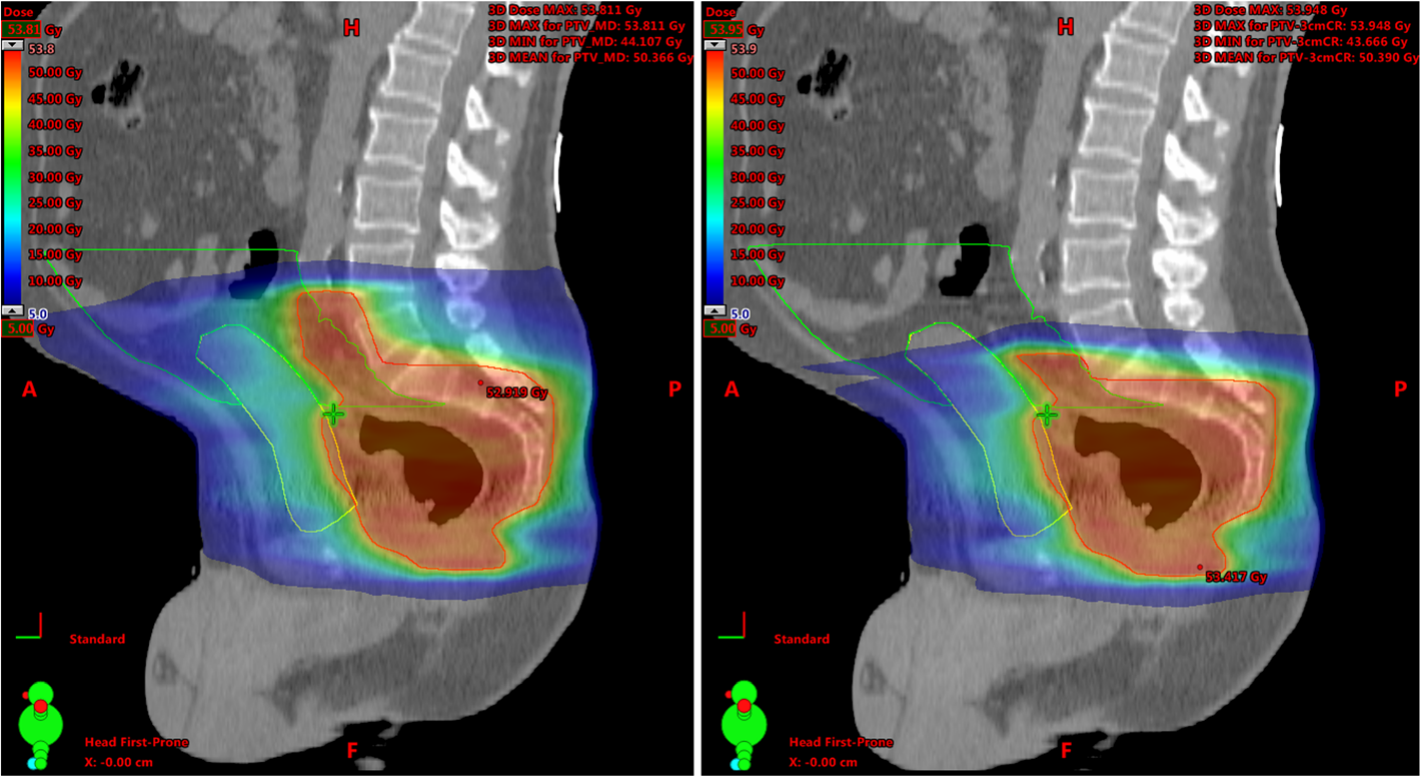
Change in dose distribution after a target volume reduction of 3 cm. Protection of OARs. Differences in dose distribution (colour-wash) of the original bPTV (left) and the cranial 3 cm reduced PTV (3 cm, right). Especially the low and middle dose range covering less volume of the peritoneal space (green shape) and the bladder (yellow shape)

The mean volumes of the PTV0, PTV-1, PTV-2 and PTV-3 were 1524 cc, 1466 cc, 1366 cc and 1255 cc. This is a reduction of just 18% from PTV0 to PTV-3. The dose coverage of all PTVs was performed accurately, which resulted in identical mean Dmean, mean Dmedian and mean Dmax. For the bone marrow, the small bowel loops and the peritoneal space nearly all absolute and relative dose parameters were significantly reduced for the new PTVs (PTV-1, PTV-2, PTV-3) compared to PTV0 (Tables 2 and 3, Fig. 2).

**Table 2:** Absolute dose parameters of organs at risk for different cranial PTVs of rectal cancer

Structure	Parameter	PTV0	PTV-1	*p*-value	PTV-2	*p*-value	PTV-3	*p*-value
		Dose (Gy)						
Rectum	Median	50.5	50.4	*0.170*	49.9	*0.220*	49.6	*0.185*
	Max	53.4	53.2	*0.110*	53.3	*0.687*	53.3	*0.624*
Sigmoid colon	Median	38.8	37.4	*0.362*	33.1	*0.126*	29.8	*0.093*
	Max	51.9	52.0	*0.575*	51.7	*0.444*	52.0	*0.221*
Femoral head	Median	23.4	23.6	*0.646*	23.9	*0.241*	23.8	*0.646*
	Max	37.9	38.2	*0.441*	38.8	*0.203*	38.6	*0.114*
Bladder	Median	28.0	27.8	*0.153*	27.0	*0.110*	**25.7**	*0.013*
	Max	52.5	52.5	*0.575*	52.7	*0.314*	52.7	*0.221*
Bone	Median	27.6	**26.6**	*0.007*	**24.9**	*0.005*	**21.9**	*0.005*
	Max	53.8	53.8	*0.959*	53.7	*0.594*	53.8	*0.959*
Small bowel loops	Median	2.7	**1.1**	*0.005*	**0.6**	*0.005*	**0.4**	*0.005*
	Max	45.5	45.5	*0.508*	42.8	*0.441*	41.1	*0.445*
Peritoneal space	Median	20.9	**15.5**	*0.005*	**9.9**	*0.005*	**5.0**	*0.005*
	Max	53.6	53.6	*0.721*	53.8	*0.386*	53.4	*0.214*
	65 cc	50.9	50.4	*0.475*	**48.5**	*0.083*	**46.1**	*0.007*
	100 cc	49.3	**48.5**	*0.153*	**46.2**	*0.028*	**44.4**	*0.005*
	180 cc	46.2	**44.2**	*0.038*	**42.8**	*0.009*	**41.6**	*0.005*
	830 cc	18.0	**14.9**	*0.012*	**9.6**	*0.012*	**7.5**	*0.012*

Bold values: statistic significant to PTV0. PTV-1 = PTV minus 1 cm on cranial site. *p*-value = PTV1,2,3 vs. PTV0

**Table 3:** Relative dose parameters of selected organs at risk for different cranial PTVs of rectal cancer

Structure	Parameter	PTV0	PTV-1	*p*-value	PTV-2	*p*-value	PTV-3	*p*-value
		Volume (%)						
Bone	V10	87	**84**	*0.007*	**78**	*0.005*	**70**	*0.005*
	V20	69	**66**	*0.005*	**62**	*0.005*	**55**	*0.005*
	V30	44	**41**	*0.007*	**39**	*0.005*	**33**	*0.005*
	V40	29	**27**	*0.017*	**24**	*0.007*	**20**	*0.005*
Darm loops	V10	21	**18**	*0.008*	**14**	*0.008*	**10**	*0.008*
	V15	19	**16**	*0.008*	**13**	*0.008*	**8**	*0.008*
	V20	14	**11**	*0.008*	**09**	*0.008*	**06**	*0.008*
	V25	9	**7**	*0.008*	**5**	*0.008*	**4**	*0.008*
	V30	6	**5**	*0.008*	**3**	*0.008*	**3**	*0.008*
	V35	4	**3**	*0.028*	**2**	*0.028*	**2**	*0.018*
	V40	3	**2**	*0.043*	**2**	*0.028*	**1**	*0.028*
	V45	2	**2**	*0.018*	**2**	*0.028*	**1**	*0.028*
	V50	1	**0**	*0.612*	**0**	*0.176*	**0**	*0.028*
Peritoneal space	V10	71	**58**	*0.005*	**47**	*0.005*	**37**	*0.008*
	V15	61	**51**	*0.005*	**42**	*0.005*	**33**	*0.005*
	V20	48	**42**	*0.005*	**34**	*0.005*	**28**	*0.005*
	V25	36	**32**	*0.005*	**27**	*0.005*	**22**	*0.005*
	V30	27	**24**	*0.005*	**21**	*0.005*	**18**	*0.005*
	V35	22	**20**	*0.005*	**17**	*0.005*	**15**	*0.005*
	V40	18	**17**	*0.005*	**15**	*0.005*	**13**	*0.005*
	V45	16	**15**	*0.005*	**13**	*0.005*	**12**	*0.005*
	V50	08	**07**	*0.114*	**06**	*0.005*	**06**	*0.005*

Bold values: statistic significant versus PTV0. PTV-1 = PTV minus 1 cm on cranial site. *p*-value = PTV1,2,3 vs. PTV0

The bone marrow had a mean volume of 1412 cc. Dmedian of the bone volume and all relative dose parameters (V10, V20, V30, V40) for all cranial shortened PTVs (PTV-1, PTV-2 and PTV-3) compared to the PTV0 were significant reduced (*p* ≤ 0.008). Relatively, the volume in low dose range didn’t decrease as much as in high dose range. The V10 of the PTV-3 (70%) was 19% less than of the PTV0 (87%) whereas the V40 was reduced by 29%. As expected, the Dmax did not change within the different plans (all 54.0 Gy).

The peritoneal space had nearly constant caudal extension to the level of S1/S2 in the different patients. The situation was similar for the small bowel. The mean volume of the peritoneal space was 1847 cc. For both, the small bowel loops and for the peritoneal space, a significant reduction of the relative dose parameters (V10, V15, V20, V25, V30, V35, V40, 45, Dmedian, Dmax) and for the peritoneal space also for the evaluated volumes (180 cc, 830 cc) was shown. The Dmedian for the peritoneal space for the PTV0, PTV-1, PTV-2 and PTV-3 was 20.9 Gy, 15.5 Gy, 9.9 Gy and 5.0 Gy (for all *p* = 0.005). The biggest relative differences in dose distribution of the peritoneal space are shown for medium-sized volumes (180 cc). Generally the dose to large volumes changed more than for very small volumes. The dose of 830 cc of the peritoneal space for the PTV-1, PTV-2 and PTV-3 was 14.9 Gy, 9.6 Gy and 7.5 Gy and all decreased significantly compared to 18 Gy of the PTV0 (*p* = 0.012). In contrast, for 65 cc, a significant dose change was just seen for PTV-3. Analogous, the largest differences in the volume of the peritoneal space were seen for low doses. The V10 of the PTV-3 was about halved compared to PTV0 while the V50 could just be reduced by about a quarter.

The mean volume of the small bowel was much higher (2977 cc) and differed much between the patients due to different cranial extensions of the planned CT imaging. This affected the Dmedian but not the relative dose parameters. Compared to the peritoneal space, a similar outcome was found for the relative dose parameters of the small bowel loops. The V10 (18%, 14% and 10%) of PTV-1, PTV-2 and PTV-3 was significant lower (for all *p* = 0.008) compared to PTV0 (21%) while there was not a big change in V50 (all 0%) compared to 1% (*p* = 0.028–0.612). The Dmax still remained high for both, small bowel loops and peritoneal space for all PTVs and did not show statistically significant changes.

A significant reduction of the Dmedian for the urinary bladder was just seen with 3 cm lowered PTV. The PTV-3 was 25.7 Gy compared to PTV0 with 28.0 Gy (*p* = 0.013) while there were no differences in different Dmax. For the sigmoid colon there was a slight decrease in dose distribution with reduced margins of the target volume (not significant) and the Dmax remained almost the same for all PTVs (about 52.0 Gy). Finally, as expected, the Dmedian and Dmax of the femoral head (left) for different PTVs did not differ significantly.

## Discussion

### Benefit of the reduction of the target volume

We could show that each cranial reduction of 1 cm from the standard PTV in rectal cancer patients of the lower and middle third can significantly reduce the dose to the bone marrow and small bowel (loops & peritoneal space) for almost all relevant dose parameters (Tables 2 and 3, Fig. 2). Also for urinary bladder the Dmedian can be reduced with a 3 cm lowered cranial margin. In similar study approaches, for example in esophageal carcinoma, it has already been shown that a reduction of longitudinal margins would probably lead to an expected lower rate of side effects [19]. So the main question is whether it is to be expected that such margin reductions can also be translated into better therapy tolerance in rectal cancer. In large prospective trials the rate of severe acute enteritis was about 15% [20, 21]. Chronical consequences of small bowel radiation can be obstruction, perforation, fistula, bleeding, persistent diarrhea and malabsorption [22, 23].

The relevance of dose-volume relationships and side effects in different types of pelvic cancer treated with chemoradiation was demonstrated. The importance of dose sparing of bone marrow was required for anal cancer [24]. For anal cancer patients treated with definitive CRT Cheng et al. could proof a highly significant correlation of ≥grade 3 hematologic toxicity with the mean dose and low-dose dose parameters (V5, V10, V15, V20) and recommended dose constraints to the lumbo-sacral-spine with V10 ≤ 80% [25]. In 50 patients treated with IMRT, a higher V20 of the pelvic bone marrow was associated with lower white blood cell nadir (*p* = 0.048) and patients with V40 ≥ 41% of the lumbo-sacral bone marrow had higher risk to develop ≥Grade 3 hematologic toxicity [26]. The most comparable study design was from Wan et al. [27]. Here the V40 of the lumbosacral spine was associated with clinically significant grade ≥ 2 hematologic toxicity in patients receiving conventional concurrent CRT (50 à 2 Gy, IMRT, Capecitabine) (grade ≥ 2hematologic toxicity with V40 ≥ 60% vs. V40 < 60% was 38.3% vs.13%, *p* = 0.005). Interestingly, no case of severe acute toxicity was registered in a radiation dose intensification study in rectal cancer [28].

In our study, V10, V20, V30 and V40 of the whole bone of the pelvis (including lumbosacral spine) would be decreased significantly with each cranial reduction of the PTV of 1 cm compared to the standard PTV (PTV0). The V10 for the bone in the pelvis (mainly lumbosacral spine) was 87% for PTV0, 84% for PTV-1, 78% for PTV-2 and 70% for PTV-3 (*p* ≤ 0.007). The effect was less but still significant in high dose range. The V40 was 29% for PTV0, 27% for PTV-1 (*p* = 0.017), 24% for PTV-2 (*p* = 0.007) and 20% for PTV-3 (*p* = 0.005). Even so the definition of the bone marrow and the type of chemotherapy could vary in those studies, with significant reduced relative dose parameters it is to be assumed that acute ≥2 and ≥ 3 hematologic toxicity would be significantly reduced as well.

The survey and evaluation of the dose distribution on the small bowel is complex. A major problem is the definition of small bowel loops on the planning CT scan because of inconsistent position of the small bowel throughout the treatment course. The evidence for relationships between dose distributions and clinical side effects is still limited, therefore clear recommendations concerning small bowel constraints are still difficult [29]. Some authors prefer the definition of the peritoneal space as a more reliable surrogate for the small bowel, whereas other studies have shown that both approaches, small bowel and peritoneal space, can be useful for dose comparison [18]. Therefore, in our study, we defined the small bowel loops and the peritoneal space. Furthermore all of our rectal cancer patients were irradiated in prone position because there is good evidence that prone position is superior to supine position in terms of dose distribution to the small bowel in patients who underwent pelvic irradiation with 3D conformal RT and most likely applies to the VMAT-IMRT as well [30, 31]. Our study design and peritoneal space definition orientated on Banerjee et al.. They have published data of 67 patients who were irradiated with nCRT and compared peritoneal space versus small bowel and evaluated dose parameters to predicting grade ≥ 3 acute toxicity. The mayor findings were, that peritoneal space V15 less than 830 cc and a small bowel V15 less than 275 cc correlated with < 10% risk of grade ≥ 3 acute toxicity [18]. In our study the volume receiving 15 Gy could be roughly halved with a PTV-reduction of 3 cm. The V15 of the peritoneal space decreased significantly with every cm. Furthermore, we had similar findings for V15 of the small bowel (551 cc, 444 cc, 339 cc and 240 cc; *p* = 0.008). Though, only the V15 of the peritoneal space for PTV-2 and -3 cm was below 830 cc and for the small bowel only the V15 of the PTV-3 was below 275 cc.

There are several mathematical models to estimate dose/volume-relationships and probability of acute side effects. Roeske et al. e. a. suggest that the volume of the peritoneal space receiving the prescription dose (45–50 Gy) should be < 195 cc [32]. As mentioned, in our study the relative changes in the dose distribution on high dose range wasn’t as high as in low dose range. In our treatment plans the V45 and V50 were 292 cc and 138 cc for the standard PTV whereas it decreased to 213 cc and 103 cc for the PTV-3 (both: *p* = 0.005). Those changes were all statistic significant. In summary of the results and the clinical relationships of the DVH and the toxicity, it should be noted that cranial target volume reduction can significantly reduce all parameters that have been clinically relevant in the literature. That might reduce acute and late intestinal toxicity but has to be proven in prospective trials which use cranially reduced CTV margins.

### The opportunity of smaller cranial margins in n(C)RT treated rectal cancer patients

The opportunity of smaller cranial margins in n(C)RT treated rectal cancer patients, especially with IMRT, of the middle and lower third is currently discussed [33]. In a recently published editorial, Te Vuong, Aurelie Garant & Fleure Gallant summarised the situation very well [34]. The TME trial could show a further reduction of local recurrence with short term neoadjuvant RT. After a median follow up of 6.1 years, RT with 5 × 5 Gy local relapse was 5.6% compared to 10.9% with TME alone [4]. Moreover RT is most effective if the quality of TME is high [35]. Next to the fact that loco-regional relapse after RT and TME is very rare, studies dealing with patterns of local relapse have shown that locoregional relapse is mainly below the level of S1-S2. In an update of the TME-trial, patterns of local relapse were published. Local relapse after RT and TME was still very rare (1.1% of all patients) and most likely posterior or laterally of the primary tumor site [36]. A Swedish study analysed the site of recurrence of rectal cancer patients treated with abdominal resection (mostly with n(C)RT). All cases of relapse (155 of 2315) have been in the lower 75% of the pelvis, means below the S1/S2 interspace [13]. Additionally, in a three-dimensional analysis of recurrence patterns in rectal cancer patients with recurrence after TME, Nijkamp et al. could demonstrate that patient without primary node involvement had no recurrences cranially of the S2-S3 interspace [14]. With CTV reduction to the S2-S3 interspace, over 60% (three-field conventional RT) and 80% (IMRT) reduction in absolute small bowel exposure (dose levels from 15 to 35 Gy) could be achieved. A cranial PTV reduction should therefore be possible without an increased risk for locoregional recurrence rates. Ongoing randomized prospective trials on the timing of surgery after IMRT-based neoadjuvant chemoradiation treatment (ClinicalTrials.gov, number NCT03465982; number NCT02551237) could provide a valid contribution to the definition of the patterns of local relapse and acute toxicity, to consolidate the clinical rationale of the reduction of the cranial target volumes for patients with rectal cancer.

## Conclusion

Reduction of the cranial target volume in nCRT for patients with rectal cancer of the lower and middle third can lead to a significant reduction of the dose parameters proven to be crucial for toxicity rates, especially acute gastrointestinal and hematologic side effects. Due to the anatomical conditions, meaningful dose restrictions to OAR that other studies have shown can best be achieved by cranial CTV reduction. Considering the very low recurrence rates in the TME and IMRT era, the distribution patterns of these relapses as well as the relevant side effects of the neoadjuvant irradiation, we would agree with existing recommendations of a reduction of the cranial target volumes at least up to the level of S1/S2 interspace.

## Abbreviations

2D/3D: 2/3 dimensional
BM: Bone marrow
BSA: Body surface area
CRT: Chemoradiation
CTV: Clinical target volume
E. a.: For example
Gy: Gray
IMRT: Intensity modulated radiotherapy
nCRT: Neoadjuvant chemoradiation
OAR: Organs at risk
PC: Peritoneal Space
PET: Positron Emission Tomography
PTV: Planning target volume
RC: Rectal cancer
RT: Radiation therapy
RT: Radiotherapie
SB: Small bowel
SIB: Simultaneously integrated boost
VMAT: Volumetric modulated arc therapy
